# Development of Low-Cost Soil Flux Chamber for CO_2_ Release Measurement

**DOI:** 10.3390/s26051602

**Published:** 2026-03-04

**Authors:** Rahul Verma, Utkarsh Prabhakar Gupta, Damar David Wilson, Venkatesh Balan, Abdul Latif Khan, Ram Lakhan Ray, Xiaonan Shan

**Affiliations:** 1 Department of Electrical Engineering, University of Houston, 4302 University Drive, Houston, TX 77004, USA; 2 Cooperative Agricultural Research Center, College of Agriculture, Food and Natural Resources, Prairie View A&M University, Prairie View, TX 77446, USA

**Keywords:** low-cost flux chamber, soil CO_2_ flux, soil respiration measurements, automated soil CO_2_ monitoring

## Abstract

Accurate measurement of soil CO_2_ flux is essential for understanding terrestrial carbon dynamics and quantifying greenhouse gas emissions from soil. However, the complexity and high cost of traditional measurement equipment limit its wide adoption in agriculture and other terrestrial ecosystems, including grasslands and managed field environments. In this paper, we developed a low-cost, automated soil CO_2_ flux chamber for soil CO_2_ flux monitoring. The flux chamber utilizes a commercially available MH-Z19 NDIR CO_2_ sensor (Winsen Electronics Technology Co., Ltd., Zhengzhou, China), integrated with a Raspberry Pi microcontroller (Raspberry Pi Ltd., Cambridge, UK; manufactured by Sony UK Technology Centre, Pencoed, Wales, UK) for automated data collection and remote monitoring. The collected data are wirelessly transmitted to a computer or mobile device for real-time display. The total material cost of the system is less than $162. Side-by-side field measurements with a commercial LI-COR 8200-01S chamber (LI-COR Biosciences, Lincoln, NE, USA) showed that CO_2_ fluxes measured by the low-cost chamber were consistently lower than those measured by the commercial instrument, averaging approximately 0.75–0.80 times the LI-COR values, indicating systematic underestimation in magnitude, while showing strong linear agreement (R^2^ ≈ 0.98–0.99) across repeated field measurements. This indicates that the system reliably tracks relative changes in soil CO_2_ flux, although a systematic bias in magnitude is present. This affordable and user-friendly chamber improves accessibility for researchers and field practitioners, enabling practical monitoring of soil CO_2_ flux in applications where cost and portability are critical.

## 1. Introduction

The CO_2_ release from soil is an important aspect of the global carbon cycle and the contributions of greenhouse gases to climate change [1]. The exchange of CO_2_ between the soil and atmosphere is caused by processes of microbial decomposition, root respiration, and breakdown of organic matter [2,3]. These soil respiration processes contribute significantly to atmospheric CO_2_ levels, influencing both ecosystem health and the global carbon budget. Therefore, the exact measurement of soil CO_2_ flux is very important for quantifying such emissions, developing strategies for carbon sequestration, and enhancing predictive climate models. However, many existing measurement systems are expensive and complex, which limits their use in routine field studies, educational settings, and projects with limited funding. Because of these constraints, there is a growing need for reliable and low-cost soil CO_2_ flux measurement systems that can provide repeatable results while improving accessibility and ease of deployment in field conditions [4,5]. CO_2_ is the most commonly emitted gas during soil respiration and plays an important role in soil carbon exchange processes [6,7]. Soil CO_2_ flux is an important indicator of soil respiration and overall ecosystem health, largely influenced by biological activity within the soil [8,9].

Soil CO_2_ flux is influenced by biological activity such as microbial decomposition and root respiration, and it varies with environmental factors including temperature, moisture, and soil structure [10,11]. Because these processes vary across sites and over time, accurate and repeatable measurement of soil CO_2_ flux is essential for understanding soil respiration dynamics and evaluating ecosystem carbon exchange [12,13,14].

Among the most widely used methods for measuring soil CO_2_ flux is the closed-chamber technique, in which a chamber is placed over the soil surface, and the increase in CO_2_ concentration is monitored over time [15,16]. The accuracy of this method depends on stable chamber conditions, reliable concentration measurements, and appropriate data analysis procedures [17].

However, many existing automated systems remain expensive and difficult to deploy widely, creating a need for reliable and accessible measurement tools that can be used in routine field studies [18]. Recent advances in chamber technologies, particularly the development of automated systems, have addressed several limitations of traditional methods by enabling continuous, high-frequency measurements [19,20,21]. Automated soil flux chambers, such as the LI-COR 8200-01S Smart Chamber Automated Soil CO_2_ Flux System (LI-COR Biosciences, Lincoln, NE, USA), offer significant improvements in data collection efficiency and accuracy [22]. These instruments are capable of measuring a variety of soil gases, including CO_2_, CH_4_, and N_2_O, with real-time data logging and customizable sampling schedules. The LI-COR 8200-01S Smart Chamber, for example, provides high-resolution measurements with a reported accuracy of approximately ±1 µmol m^−2^ s^−1^ for CO_2_ flux, as specified by the manufacturer [23,24,25]. This accuracy specification applies to the commercial instrument and not to the low-cost chamber developed in this study. The system is designed to provide reliable results under a wide range of environmental conditions [26,27]. It also integrates sensors for ambient environmental monitoring, such as soil temperature and moisture, further enhancing the quality and context of the collected data.

Despite their technological advancements, automated chambers still come with higher costs, often ranging from $20,000 to $50,000 or more, depending on the model and configuration. This price tag makes them inaccessible for many researchers, particularly those working with limited funding. Additionally, these systems tend to be complex and require regular calibration, maintenance, and expert handling to maintain accuracy [28]. Operational challenges also arise in field applications, particularly in remote locations where power sources may be unreliable and environmental conditions are highly variable [29,30]. Moreover, the adaptability of these instruments to different soil types, vegetation cover, or extreme weather conditions remains a challenge, limiting their universal applicability. Given these limitations, long-term ecological studies and remote monitoring projects still face significant hurdles [31,32,33]. This emphasizes the need for more cost-effective, adaptable, and user-friendly soil CO_2_ flux measurement technologies that can integrate into diverse field conditions while maintaining high data quality [34,35].

Existing studies have explored simplified or low-cost soil CO_2_ measurement systems, but many of these approaches either lack automated operation, are not quantitatively validated against commercial instruments, or do not report clear performance metrics such as repeatability, regression quality, and measurement uncertainty under field conditions [35,36]. As a result, there remains a need for low-cost systems that are not only affordable but also systematically evaluated using measurable performance criteria [37,38].

The objective of this study is therefore to design and evaluate a low-cost automated soil CO_2_ flux chamber and to quantify its performance using field measurements. Specifically, we evaluate (1) agreement with a commercial LI-COR chamber using percent difference and linear regression metrics, (2) repeatability through replicated measurements, and (3) performance across different soil and environmental conditions. Our working hypothesis is that a low-cost automated chamber can provide sufficiently accurate relative flux measurements for short-term field studies while significantly reducing system cost and complexity [39,40,41].

In this study, we developed a low-cost automated soil CO_2_ flux chamber to support repeatable field measurements at a much lower cost than commercial systems. The system uses a low-cost NDIR CO_2_ sensor with automated data logging and a simple reset step between measurements. Full details on system components, operation, and flux calculation are provided in Section 2.

The system design, operational workflow, and data acquisition procedures are described in Section 2, followed by experimental results and performance evaluation. We assess the capabilities of our soil flux chamber across a range of soil types and field conditions, including a side-by-side evaluation with a commercial soil flux chamber conducted in a farmland setting. The results indicate that the device reliably captures trends over time in soil CO_2_ flux and provides repeatable measurements suitable for comparative field studies. Notably, the total material cost of the device is under $162. This system is reliable, efficient, and adaptable for comparative soil respiration measurements in field studies where cost and portability are important.

## 2. Materials and Methods

### 2.1. System Components and Hardware Integration

In this paper, we developed a low-cost soil CO_2_ flux measurement chamber that overcomes many of these limitations. Our system is built on a nondispersive infrared (NDIR) CO_2_ sensor for real-time measurements of the CO_2_ concentration in a chamber fixed on top of the soil of interest. A 3D-printed chamber is used to collect the gases released from the soil. The CO_2_ sensor is controlled, and the data is collected by a Raspberry Pi microcontroller (Raspberry Pi Ltd., Cambridge, UK; manufactured by Sony UK Technology Centre, Pencoed, Wales, UK) that enables automated operation. The collected data were converted to CO_2_ flux based on the chamber dimensions, and the results were wirelessly transmitted and displayed on the computer. In addition, we significantly lowered costs by removing the costly mechanical actuators for chamber operation, instead utilizing an economical pump to allow for efficient air circulation within the chamber. This design not only promotes thorough gas mixing for precise CO_2_ measurements but also streamlines the system’s mechanical complexity. A relay controls the pump, enabling automated adjustments in response to sensor data, all controlled through the Raspberry Pi platform. Through a computer interface, users can easily adjust pump parameters and oversee system performance, improving ease of use.

The design of the chamber meets the requirements for providing a consistent and reliable measurement of the flux of CO_2_ by addressing some challenges in the traditional system. An automated pump that resets the CO_2_ levels enables continuous testing, avoiding most of the errors related to the opening and closing of the chambers. Continuous data logging of the CO_2_ concentrations is also provided by the system, which may enable real-time flux calculations of high accuracy. The system design addresses common challenges in closed-chamber measurements, including gas mixing and repeatable measurement cycles. Automated pump operation allows the chamber to be reset between measurements without manual intervention, and continuous data logging enables repeatable flux estimation. Its real-time data, automated functionality, and low maintenance enable repeatable soil CO_2_ flux measurements suitable for field studies, educational use, and preliminary soil respiration assessments.

The low-cost soil CO_2_ flux chamber uses a single 3D-printed part in which the collar and chamber body are printed together as one unit (PLA). The soil opening diameter is 20 cm, which defines the soil surface area used in flux calculations. The MH-Z19 NDIR CO_2_ sensor (Winsen Electronics Technology Co., Ltd., Zhengzhou, China) was mounted near the top of the chamber headspace to measure CO_2_ concentration. Soil temperature was measured using a DS18B20 sensor (Maxim Integrated, San Jose, CA, USA) and soil moisture was measured using an LM393 sensor (generic supplier, Shenzhen, China). A diaphragm pump (R385 diaphragm pump; ZWDZ, manufactured in China; sourced via Amazon, Seattle, WA, USA) was connected to the chamber via tubing and controlled through a relay module. All electronics were housed in a sealed controller box next to the chamber. The system was battery-powered for field use.

The geometric and construction parameters used for flux calculations are summarized in Table 1, including soil opening diameter, surface area, insertion depth, chamber height above soil, headspace volume, wall thickness, and sealing method. Insertion depth and chamber height were measured in the field during installation.

### 2.2. Control Logic and Data Acquisition

The Raspberry Pi 4 ran a Python program (Python 3.14.3; Python Software Foundation, Wilmington, DE, USA) on Raspberry Pi OS (Raspberry Pi Ltd., Cambridge, UK) to control the measurement cycle, read sensors, and save data. CO_2_ was read from the MH-Z19 over UART and recorded with timestamps at a sampling interval of 1 s. Each measurement cycle used a 120 s chamber closure (pump OFF) followed by a reset step where the pump was turned ON until CO_2_ returned near baseline (typically 30–40 s with the higher-flow pump). Data were saved as CSV files containing timestamp, CO_2__ppm, soil_temp_C, soil_moisture_raw, and pump_state. Files were transferred to a computer over Wi-Fi for plotting and offline flux calculation. Data processing and visualization were performed using Python libraries including NumPy, Pandas, and Matplotlib.

### 2.3. Flux Calculation

CO_2_ flux was calculated using a closed-chamber approach. CO_2_ concentration (ppm) was converted to molar fraction by dividing by 10^6^. The rate of change was estimated using a linear fit of CO_2_ concentration versus time. To reduce early mixing effects after closing the chamber, we excluded the first 20 s and fit the slope using 20–120 s after closure. We report R^2^ for the fit and only used segments that showed a clear linear increase.

### 2.4. Data Communication and User Interface

Data were transmitted wirelessly to a computer using Wi-Fi. A Python-based interface enabled users to start/stop measurements and view CO_2_ concentration in real time for quality checking. Following data collection, CO_2_ flux was computed offline by fitting a linear regression to CO_2_ concentration versus time over the defined closure window and using the resulting slope in the closed-chamber flux equation described in the manuscript. This workflow enables repeatable soil CO_2_ flux measurements with minimal user intervention.

## 3. Results and Analysis

### 3.1. Low-Cost Soil Flux Chamber Design

In order to achieve a low-cost soil CO_2_ flux measurement, we begin with the design of the chamber. A conventional soil CO_2_ flux chamber includes several parts: a base and seal to secure it into the soil, a metal chamber dome or cover to trap gases, a gas collection system to transport gases, a sensor housing for gas analysis, and a data logger with a power supply for continuous measurement [42]. These components make the chamber quite expensive. The costliness stems from both the high-quality materials needed for the chamber itself and the sophisticated systems required to collect and analyze the gas using high-performance sensors.

On the other hand, there are commercially available CO_2_ sensors that are sensitive enough to measure the CO_2_ emissions directly from the soil. These sensors provide a more affordable alternative by simplifying the traditional setup. By incorporating these sensors directly into the chamber design, we can eliminate the need for costly gas transfer systems and high-end sensor cases, significantly reducing the overall cost while maintaining functionality for accurate measurements.

**Figure 1:**
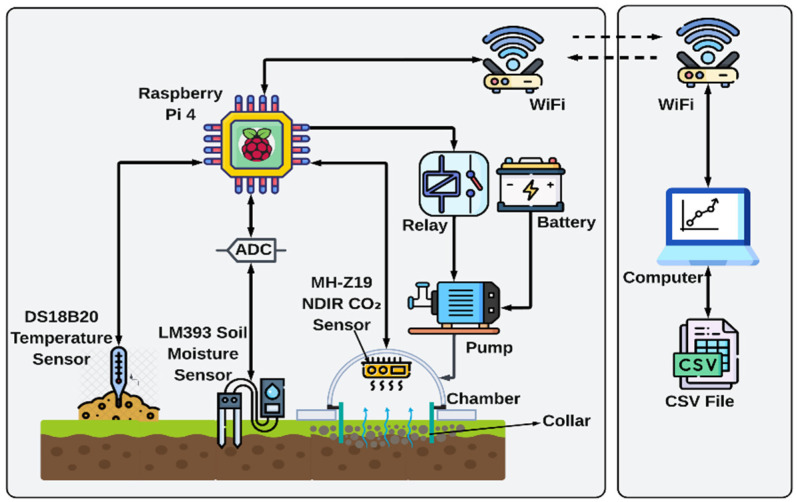
Low-cost soil CO_2_ flux chamber diagram.

Therefore, we designed our soil CO_2_ flux chamber as shown in Figure 1. Instead of using a high-accuracy and high-cost gas transfer NDIR sensor, we use the low-cost, commercially available MH-Z19 NDIR CO_2_ sensor to measure the CO_2_ concentration. This sensor costs approximately $25 and is also readily available. The price can be even lower if a large number of sensors are ordered. In addition, to minimize costs, we do not transfer the gas released from the soil to a separate flux analyzer. Instead, we have positioned the sensor directly inside the chamber to perform on-site measurements. The CO_2_ sensor is controlled by a Raspberry Pi 4, and the same Raspberry Pi collects the data via UART communication, which is a low-cost controller for IoT applications. After collecting data, we use Wi-Fi to transfer the data to the computer and display the result in real time. Furthermore, to measure soil temperature and soil moisture, we have also integrated the DS18B20 temperature sensor and the LM393 soil moisture sensor into our system [43]. The operational configuration and calibration considerations for the CO_2_ sensor are described in a later subsection. Low-cost NDIR sensors such as the MH-Z19 have known limitations, including sensitivity to temperature and humidity, long-term drift, and automatic baseline calibration behavior that can introduce bias in enclosed environments. These factors were considered in the design and operation of the system and are discussed in detail in Section 3.5.

Furthermore, we also significantly reduced costs by eliminating the need for expensive mechanical actuators to open and close the chamber; we used a low-cost pump to circulate air effectively within the chamber. This approach not only ensures proper gas mixing for accurate CO_2_ measurements but also simplifies the mechanical design. The pump is controlled with a relay, which allows for automated control based on sensor feedback, and this setup is managed via the Raspberry Pi system. Users can easily adjust pump settings and monitor the system through a user-friendly computer interface, enhancing operational convenience.

**Figure 2:**
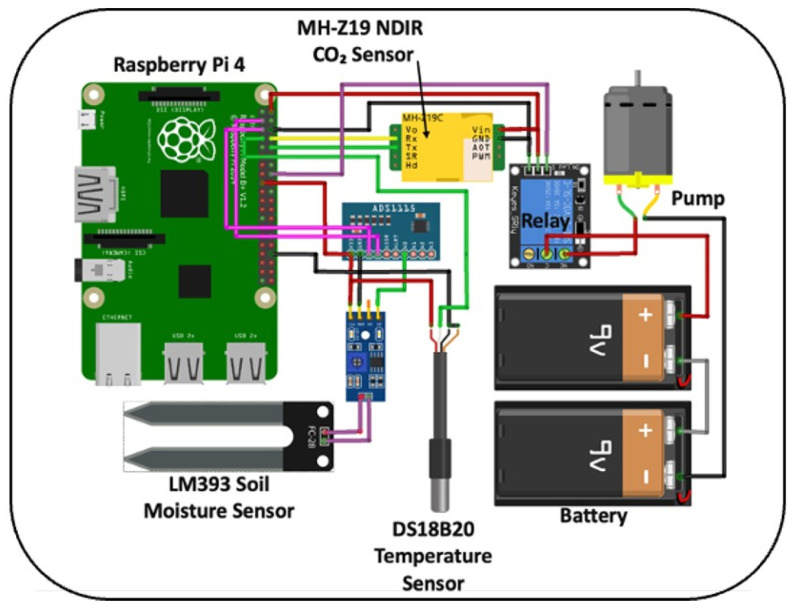
Low-cost soil CO_2_ flux circuit diagram.

The entire system is powered by a battery, ensuring that it can operate in field conditions without requiring access to electrical outlets. This portability allows for flexibility in testing different soil types in various locations. To initiate a new test, users can simply activate the pump via the software, which is designed to start and stop the pump with minimal user intervention. Figure 2 shows the circuit diagram and the connections between devices and peripherals, providing a clear guide for setup and troubleshooting.

In addition to utilizing low-cost CO_2_ sensors and a data collection system, we have also used generic, off-the-shelf electronic components to further decrease costs. Table 1 summarizes the material cost of the entire system. Each component is listed together with unit cost, quantity, and total cost contribution to improve transparency and reproducibility. Only material costs are included in the total; labor, printer depreciation, and assembly time are not included.

The chamber body and collar were fabricated using PLA filament on a 3D printer (Bambu Lab, Shenzhen, China). The filament material cost is included in Table 1 as part of the total system cost. With all material costs included, the total material cost of the system, including sensors, electronics, pump, and 3D-printed chamber materials, is less than $162. The detailed material cost breakdown of individual components is presented in Table 2.

### 3.2. Fabricated Soil Flux Chamber

**Figure 3:**
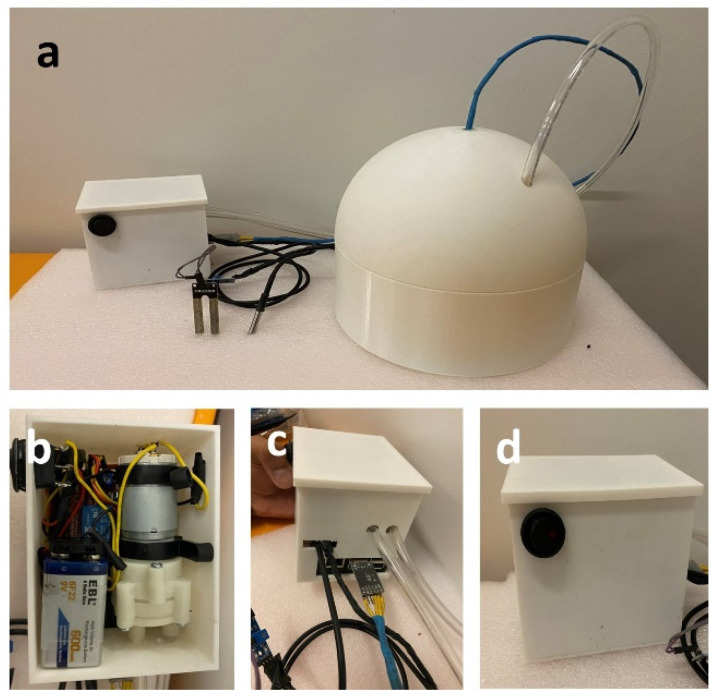
Low-cost soil flux chamber prototype. (**a**) Complete assembled soil flux chamber setup; (**b**) Internal enclosure showing pump, battery, and electronic components; (**c**) Tubing connections and sensor wiring interface; (**d**) External enclosure view with gas inlet/outlet port.

Figure 3 shows the soil CO_2_ flux chamber system we developed. To reduce costs, we utilized a 3D-printed construct for both the chamber and its collar. The chamber is designed with a diameter of 20 cm to accommodate sufficient soil volume while maintaining compactness. We intentionally made the chamber walls 2 mm thick to prevent any gas leakage, ensuring that our measurements are accurate and reliable [44]. The main setup features a spherical chamber with a polymer tube extending from the top, used to pump fresh air into the chamber when there is a need to start a new measurement. Next to it, the controller box, which has essential electronics and a microcontroller, and it connects via a blue cable to the MH-Z19 NDIR CO_2_ sensors inside the chamber [45].

In the controller box, one can see the arrangement of a Raspberry Pi 4 along with other electronics, including a pump that helps circulate air from outside to inside the chamber, which is controlled via a relay. The back view of the control box displays the various cable connections that enable power supply, temperature sensor, and moisture sensor connections. The front of the controller box includes a single user-interface button, simplifying the operation of the system. This setup allows for easy initiation or resetting of measurement processes with minimal interaction.

This design not only keeps the material costs low but also integrates sophisticated data-handling capabilities directly linked to the Raspberry Pi, enabling real-time data analysis and adjustments. The soil moisture and temperature sensors are directly connected to the Raspberry Pi 4 in the controller box, enhancing the accuracy and responsiveness of the system to environmental changes. To ensure the integrity of our measurements, special attention was given to the sealing of cables and tubes coming out of the chamber, effectively minimizing potential gas leaks and external air interference. Leak testing and pressure behavior verification are described in a later subsection.

### 3.3. Principle Demonstration

**Figure 4:**
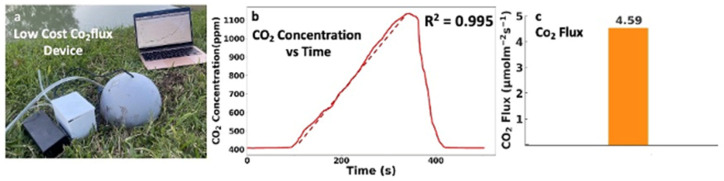
Low-cost soil CO_2_ flux chamber and representative measurements. (**a**) Photograph of the deployed low-cost soil CO_2_ flux chamber in the field. (**b**) CO_2_ concentration inside the chamber as a function of time after chamber closure. The solid line represents measured CO_2_ concentration, and the linear increase used for flux calculation shows strong linearity (R^2^ = 0.991). (**c**) Calculated soil CO_2_ flux derived from the slope of the linear regression of CO_2_ concentration versus time.

To evaluate the performance of our low-cost soil CO_2_ flux chamber, we conducted a field test. Figure 4a illustrates our device, which is embedded in the soil to monitor CO_2_ emissions resulting from microbial decomposition, root respiration, and organic matter breakdown. We inserted the one-piece, 3D-printed chamber collar unit into the soil to create a sealed headspace above the soil surface.

Initially, the CO_2_ concentration inside the chamber is around 400 ppm, which is typical for atmospheric air. Once the chamber is sealed, CO_2_ generated by the soil is trapped and begins to accumulate. The increase in CO_2_ is continuously measured by a sensor inside the chamber, which sends the data to a controller box via UART communication. This real-time data transfer allows us to monitor the rising CO_2_ levels on a computer, as depicted in Figure 4a.

Figure 4 shows a representative field test. After the chamber was inserted into the soil and sealed, the CO_2_ concentration increased over time. The selected analysis window showed strong linear behavior (example: R^2^ = 0.995). After the reset step, CO_2_ returned near baseline, enabling repeated measurement cycles.

After gathering the data, the rate of change in CO_2_ concentration is obtained by linear regression of CO_2_ concentration (ppm) versus time (s). The slope of this regression is used as Δ*C*/Δ*T* in the flux calculation.

To reduce the effect of mixing immediately after chamber closure, the slope is calculated using data from 20 to 120 s after chamber closure. The first portion of the record is excluded because transient mixing and stabilization can produce non-linear behavior. Only time intervals showing a clear linear increase in CO_2_ concentration are used. Data segments showing obvious non-linear trends, such as those affected by mixing disturbances, leakage, or sensor instability, are excluded from analysis.

Ordinary least-squares linear regression is applied to estimate the slope. Robust fitting methods were not required because the selected time window consistently showed linear behavior under field conditions. The resulting slope value is then used to calculate CO_2_ flux using the equation below [26].

For each measurement round, the goodness-of-fit of the linear regression was evaluated using the coefficient of determination (R^2^). Only time windows showing strong linearity were used for flux estimation. For the representative example in Figure 4b, the linear regression over the 20–120 s window yields R^2^ = 0.995.

$${CO}_{2}\;Flux=\frac{PV}{SRT}\cdot \frac{\Delta C}{\Delta T}$$

where

- *P* stands for the atmospheric pressure (101,325 Pa).
- *V* represents the volume of air inside the chamber, including the collar above the soil.
- *S* is the surface area of the collar.
- *R* is the universal gas constant (8.3144598 m^3^·Pa·K^−1^·mol^−1^).
- *T* is the air temperature inside the chamber in Kelvin. In this study, chamber air temperature was assumed to be close to ambient temperature during the short measurement period.
- Δ*C*/Δ*T* is the rate of change in CO_2_ molar fraction over time, obtained from linear regression of concentration versus time after unit conversion.

CO_2_ concentration is measured by the MH-Z19 sensor in parts per million (ppm). Before calculating flux, the concentration values are converted to molar fraction by dividing by 10^6^. In the flux equation, *C* is therefore treated as a molar fraction rather than ppm, and Δ*C*/Δ*T* represents the rate of change in molar fraction with time. Flux values calculated from this equation are obtained in mol m^−2^ s^−1^ and are reported in µmol m^−2^ s^−1^ by multiplying by 10^6^.

**Figure 5:**
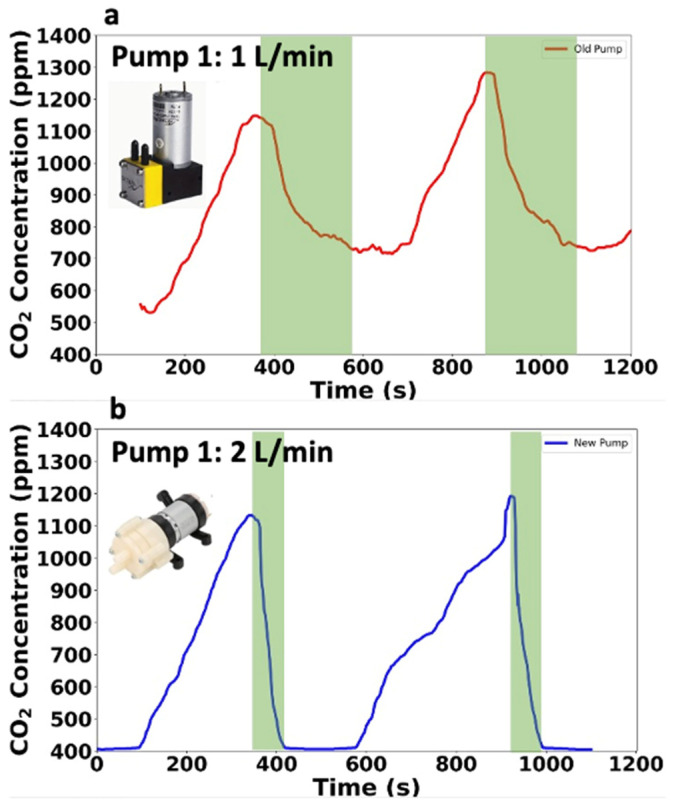
Pump Comparison. (**a**) CO_2_ concentration over time using Pump 1 (1 L min^−1^); (**b**) CO_2_ concentration over time using Pump 2 (2 L min^−1^).

**Figure 6:**
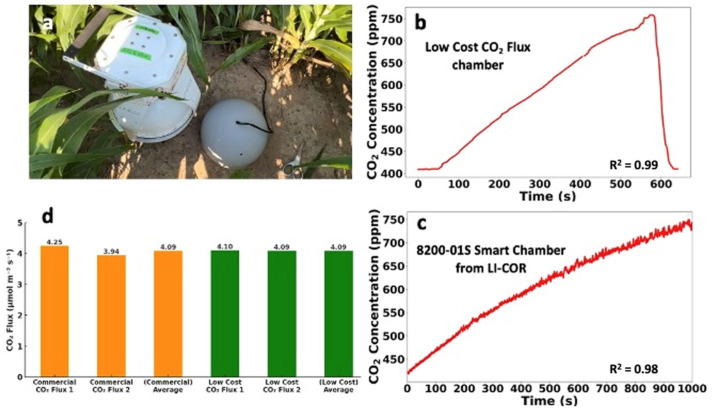
Comparison of the low-cost soil CO_2_ flux chamber with a commercial system in a cornfield. (**a**) Field deployment of the low-cost chamber during measurement; (**b**) CO_2_ concentration response over time using the low-cost chamber; (**c**) CO_2_ concentration response over time using the LI-COR 8200-01S smart chamber; (**d**) Comparison of CO_2_ flux measurements between the commercial system and the low-cost chamber.

Atmospheric pressure *P* is assumed constant and set to 101,325 Pa, which is appropriate for the field conditions under which the measurements in Figure 4 and Figure 5 were collected. The comparison with the commercial system is shown later in Figure 6. Pressure was not measured independently during these tests, and no rapid pressure changes were observed during the measurement periods.

The chamber headspace volume *V* is defined as the total air volume enclosed above the soil surface, including both the chamber body and the portion of the collar extending above the soil. This geometry is illustrated in Figure 1 and Figure 3, and the same headspace volume definition is used consistently for all flux calculations reported in this study.

Air pressure and air temperature are variables that can influence flux calculations in closed-chamber measurements. In commercial systems, these quantities are often measured directly using dedicated sensors. In the present low-cost design, a separate air pressure or air temperature sensor was not included in order to maintain system simplicity and reduce cost.

Atmospheric pressure was therefore assumed constant and set to standard ambient pressure (101,325 Pa) during the short chamber closure period of approximately 120 s, over which pressure variations are expected to be small. In addition, the chamber includes a pressure release tube that allows slow equilibration with ambient air, reducing pressure buildup caused by chamber placement or air circulation.

A soil temperature sensor integrated into the system was used to monitor environmental conditions, but soil temperature was not used directly as air temperature in the flux equation. Because measurements were short and conducted under relatively stable field conditions, the chamber air temperature was assumed to be close to ambient temperature. Because the chamber closure period was short (≤120 s), temperature changes inside the chamber during a single measurement were assumed to be negligible. This simplified approach may introduce some uncertainty and represents a limitation of the current low-cost system. Environmental temperature conditions were monitored during field measurements to verify that temperature variations were small during the short chamber closure period.

This formula enables us to estimate the rate at which CO_2_ is being released from the soil. By applying this equation, we calculate the CO_2_ fluxes, which are shown in Figure 4c. This graph illustrates the CO_2_ flux during the testing period, providing a clear visualization of the dynamics of CO_2_ release under natural conditions.

### 3.4. Leak and Pressure Verification

To maintain stable chamber conditions during measurements, the chamber was designed to minimize pressure disturbances and leakage. Pressure equilibration is achieved through the air circulation path formed by the pump and tubing, as illustrated in Figure 1 and Figure 3. During flux measurements, the pump remains off and operates only during the chamber reset stage. This prevents pressure buildup during accumulation periods. The chamber is not sealed to withstand pressure differences, and the tubing path allows slow equilibration with ambient pressure while still limiting gas exchange.

A simple leak check was performed using a CO_2_ decay test. After raising the chamber CO_2_ concentration to a known level, similar to the conditions shown in Figure 4b, the chamber was left sealed without soil flux contribution. The CO_2_ concentration changed by less than 1–2% over the observation period, which is substantially smaller than the accumulation slopes observed during flux measurements. This corresponds to a decay rate substantially lower than the accumulation slopes observed during flux measurements, indicating negligible leakage under measurement conditions. This confirms that the concentration increases observed during field measurements are primarily due to soil emissions rather than chamber leaks.

All cable and tubing penetrations through the chamber wall were sealed using epoxy and tight-fitting grommets, as described in the Chamber Fabrication Section and shown in Figure 3. These seals reduce external air intrusion and help maintain stable internal conditions during measurements.

### 3.5. Sensor Calibration and Uncertainty Analysis

To ensure reliable measurements, calibration and operational settings of the CO_2_ sensor were carefully considered. The MH-Z19 sensor includes an automatic baseline calibration (ABC) function that assumes periodic exposure to fresh outdoor air [46]. Because this assumption does not hold for a closed soil flux chamber, the ABC function was disabled during all field measurements to prevent unintended drift in concentration readings.

The sensors used in this study were factory-calibrated according to the manufacturer’s procedure prior to use. In addition, sensor performance was evaluated through side-by-side field testing with a commercial LI-COR 8200-01S soil CO_2_ flux chamber (LI-COR Biosciences, Lincoln, NE, USA). The CO_2_ concentration trends closely matched those measured by the LI-COR system, and flux values showed strong linear agreement, although our chamber produced systematically lower flux magnitudes.

Since CO_2_ flux is calculated from the rate of change in concentration inside the chamber, measurement uncertainty in CO_2_ concentration propagates directly into the calculated flux. Because flux is derived from the slope of CO_2_ concentration versus time, uncertainty in concentration measurements contributes to uncertainty in the estimated slope and therefore in flux. To first order, relative error in concentration measurements produces a similar order of uncertainty in the calculated flux, although regression fitting and sensor noise also contribute. To minimize additional variability, the CO_2_ sensor was fixed in position at the top of the chamber using epoxy, ensuring stable placement and consistent air sampling during measurements. Locating the sensor inside the chamber also avoids losses and delays associated with gas transfer tubing, reducing another potential source of measurement error.

### 3.6. Pump Optimization

To enhance accuracy in our soil flux chamber tests, we initially used a diaphragm pump. This pump had a flow rate of 0.4 to 1 L/min with a pressure of 1 bar and operated on 24 volts, requiring four batteries. However, its lower air flow rate was insufficient to quickly fill the chamber with fresh air, and it struggled to return to the initial CO_2_ concentration, as illustrated by the green bar in Figure 5a, which shows prolonged pump-down times and an inability to reach the starting CO_2_ levels.

Furthermore, the pump was relatively expensive at $27, and the high battery requirement increased operational costs and complexity. Therefore, we switched to a new pump offering a higher air flow rate of 1.5 to 2 L/min, costing only $10. These two pump configurations are shown in Figure 5 (low-flow pump in Figure 5a; higher-flow pump in Figure 5b). This more efficient pump operates on 12 volts and requires two batteries instead of the four used by the old pump, making it both cost-effective and energy efficient. Figure 5 compares the performance of these two pumps. Figure 5a corresponds to the low-flow pump (0.4–1 L/min), and Figure 5b corresponds to the higher-flow diaphragm pump (1.5–2 L/min). It highlights that, with the old pump, even after 200 s, the CO_2_ level could not return to the baseline. In contrast, the new pump resets the CO_2_ levels to the baseline within 30–40 s. Besides being faster, the new pump is also more compact and lightweight, needing only two batteries to operate effectively. As a result, we incorporated the new diaphragm pump into our system to enhance efficiency, accuracy, and affordability in our design.

### 3.7. Comparison with the Commercial 8200-01S Smart Chamber from LI-COR and Our Device

To validate the performance of our low-cost soil CO_2_ flux chamber, we conducted comparative testing alongside a commercial device, the 8200-01S Smart Chamber from LI-COR. This test was carried out in the cornfields of Prairie View A&M University (PVAMU). As shown in Figure 6a, both devices were placed side by side to ensure accuracy in comparison.

The comparison was conducted at a single agricultural field site (cornfield at Prairie View A&M University, Texas) under uniform soil and surface conditions. Both chambers were installed using collars with the same inner diameter (20 cm) to ensure an identical soil surface area. The two chambers were placed approximately 2–5 cm apart to sample nearly identical soil conditions while avoiding physical contact. Multiple measurement rounds were conducted at the same location to evaluate repeatability.

For the low-cost chamber, each measurement round used a chamber closure period of 120 s, and CO_2_ flux was calculated from the linear slope of CO_2_ concentration versus time as described in the previous section. The LI-COR 8200-01S Smart Chamber (LI-COR Biosciences, Lincoln, NE, USA) was operated using its standard automated measurement cycle following the manufacturer’s recommended settings, and flux values were computed using the LI-COR internal closed-chamber algorithm.

Our primary goal was to assess the precision of our system by comparing its CO_2_ measurement capabilities with those of the LI-COR device [47]. Figure 6b presents the CO_2_ concentration readings from our low-cost device, while Figure 6c shows readings from the 8200-01S Smart Chamber. Both figures illustrate a similar trend in CO_2_ concentration, which linearly increases over time. For both systems, we fit a linear regression to the selected post-closure window and report the corresponding R^2^ values in Figure 6 as a quality indicator for the flux calculations. This similarity in data trends supports the reliability of our device in tracking CO_2_ flux in soil. The measurement configuration used for the side-by-side comparison is summarized in Table 3.

Further, we conducted multiple test rounds to compare the CO_2_ flux results more extensively, as shown in Figure 6d. The orange bar represents the CO_2_ flux measurements from the LI-COR chamber, and the green bar indicates the flux from our device. The results showed that CO_2_ flux values measured by the low-cost chamber were within approximately 0.75–0.80 times the commercial measurements, with linear agreement (R^2^ ≈ 0.98–0.99). These tests confirm that the low-cost chamber reliably tracks relative changes in soil CO_2_ flux and shows strong linear agreement with the commercial instrument, although a systematic bias in magnitude is present. These results demonstrate the potential of the device as a low-cost alternative for comparative soil respiration measurements in field studies.

Although the trends and average flux values from the low-cost chamber closely matched those obtained from the LI-COR system, a full quantitative accuracy calibration was beyond the scope of this study and would require controlled experiments under laboratory reference conditions. Instead, system performance was evaluated through side-by-side field measurements and analysis of the linear regression fits used in flux calculation, which consistently showed stable linear behavior during the selected time windows. These results indicate that the system provides reasonable accuracy for field soil CO_2_ flux measurements, particularly in comparative or monitoring applications. However, the device is not intended to replace high-precision commercial instruments where absolute accuracy under controlled conditions is required.

### 3.8. CO_2_ Flux Measured in Different Soil Conditions

To explore how various environmental conditions affect CO_2_ release from soil, we conducted tests using our low-cost soil CO_2_ flux chamber in different settings [46]. CO_2_ release in soil is primarily driven by microbial decomposition, root respiration, and the breakdown of organic matter. Soil temperature and soil moisture were measured simultaneously during each flux measurement using the integrated sensors described earlier, allowing environmental conditions to be recorded together with CO_2_ flux. Factors such as temperature can significantly influence microbial activity and, consequently, CO_2_ flux [47].

**Figure 7:**
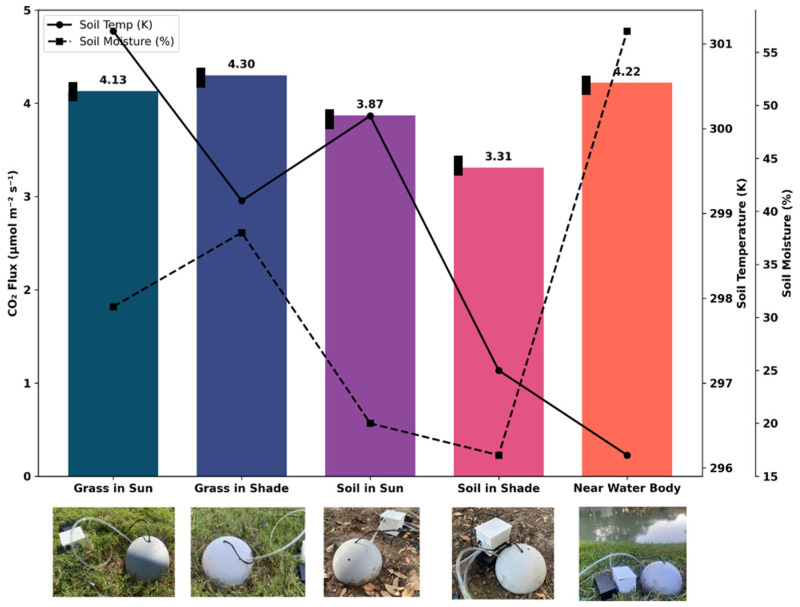
CO_2_ flux, soil temperature (K), and soil moisture (%) across different soil conditions. Error bars represent the standard deviation of replicated measurements (*n* ≥ 3).

In our study, we compared the CO_2_ flux in several soil conditions: soil exposed to sunlight versus soil in shade, grassy soil in sunlight compared to grassy soil in shade, and soil located near a water body. Repeated measurements were performed at each site under similar conditions to evaluate repeatability. These different conditions, together with the recorded soil temperature and moisture values, provide insights into how environmental variables affect the soil’s carbon dynamics. During these measurements, soil temperature and moisture varied across the test conditions. Grass-covered soil showed higher moisture and slightly lower temperature compared with bare soil, while soil near the water body had the highest moisture levels. The measured temperature and moisture values for each condition are now included in Figure 7 to provide an environmental context for the observed differences in CO_2_ flux. Figure 7 presents a bar chart comparing the CO_2_ flux across these various conditions. Each bar represents the mean value from repeated measurements (*n* > 3), and error bars indicate 1 × standard deviation. The results show that grassy soil typically shows higher CO_2_ flux than bare soil, indicating more active microbial processes in grass-covered areas. This is likely due to the enhanced organic matter and root activity in grassy soil, which provides more resources for microbial activity. These findings highlight the versatility and effectiveness of our portable soil CO_2_ flux chamber in capturing and recording CO_2_ flux across diverse environmental conditions. The data reinforce our device’s reliability and its potential as a valuable tool for soil health assessment.

### 3.9. Limitations and System Deficiencies

While the proposed low-cost soil CO_2_ flux chamber demonstrates consistent performance and strong linear agreement with a commercial system under the conditions tested, several limitations should be acknowledged.

First, the system does not include high-precision air pressure and air temperature sensors inside the chamber. Commercial chambers directly measure these variables to correct flux calculations. In our design, measurements are conducted over a short closure period (typically < 120 s), during which pressure changes are small. To further reduce pressure buildup, a pressure release tube was added to the chamber. The tube is long enough to prevent backflow while allowing excess pressure to equilibrate with ambient air. This low-cost approach reduces pressure-related artifacts but may still introduce small uncertainty compared to fully instrumented commercial systems.

Second, the CO_2_ sensor used (MH-Z19) is a low-cost NDIR sensor with lower absolute accuracy than research-grade analyzers. Although our side-by-side comparison with the LI-COR 8200-01S shows strong agreement in trends over time, a systematic difference in flux magnitude is present, and small sensor drift and noise may affect long-term measurements.

Third, flux calculations rely on linear regression of CO_2_ concentration versus time. This approach assumes a linear increase during chamber closure, which is valid for short measurement intervals but may deviate during longer closure times due to gas accumulation, diffusion limits, or leakage. To address this, we report R^2^ values for each regression and exclude non-linear portions of the curve from flux calculation.

Fourth, although replicated measurements (*n* > 3) were performed at each site and error bars are now included in Figure 7, the number of sites and soil types tested remains limited. Additional measurements across different seasons, soil textures, and moisture conditions would further improve confidence in system robustness.

Finally, compared to commercial systems, the chamber does not actively control chamber temperature or humidity. Environmental variability may therefore influence measurements, particularly under extreme field conditions.

Overall, these limitations reflect trade-offs made to achieve a low-cost, portable, and easy-to-use system. Despite these constraints, the device provides reliable relative flux measurements and is well-suited for educational use, preliminary field surveys, and applications where cost and accessibility are critical. These limitations are common in low-cost chamber designs and represent trade-offs between cost, portability, and metrological precision.

## 4. Conclusions

In this study, we designed and evaluated a low-cost automated soil CO_2_ flux measurement system based on an MH-Z19 NDIR sensor and a Raspberry Pi controller. The system enables automated measurements, wireless data logging, and field operation at a material cost substantially lower than that of commercial instruments. The system is most appropriate for short-duration measurements and comparative studies where portability and cost are prioritized over absolute metrological precision.

Field tests showed that the device is capable of capturing changes in CO_2_ concentration and estimating relative soil CO_2_ flux across different soil conditions. Side-by-side comparisons with a commercial LI-COR 8200-01S chamber showed similar trends and consistent linear behavior during flux estimation, indicating that the system can provide repeatable and practically useful measurements for comparative field studies and applications.

At the same time, several limitations should be considered. The system does not include high-precision air pressure or air temperature measurements, and the low-cost CO_2_ sensor may be affected by humidity, condensation, temperature variation, and long-term drift. These factors may introduce uncertainty, particularly during extended deployments or under rapidly changing environmental conditions. For this reason, the current design is most suitable for short measurement campaigns, exploratory studies, educational use, and applications where relative comparisons are sufficient rather than for high-precision long-term monitoring.

Further work is needed to strengthen the metrological characterization of the system. Future validation should include laboratory calibration using reference gas mixtures, multi-day stability testing to quantify sensor drift, evaluation under varying pressure and temperature conditions, and controlled leak testing. These steps would allow more rigorous estimation of accuracy, precision, detection limits, and the influence of chamber closure time.

Overall, the results demonstrate that a low-cost automated chamber can provide practical and repeatable soil CO_2_ flux measurements within the tested range of conditions. Such systems may help expand access to soil respiration monitoring in situations where commercial instruments are not available while recognizing the trade-offs between cost, precision, and long-term stability.

## Figures and Tables

**Table 1:** Geometric and construction parameters of the low-cost soil CO_2_ flux chamber used in flux calculations.

Parameter	Value	Description
Soil opening diameter	20 cm	One-piece printed chamber
Surface area (S)	0.0314 m^2^	Calculated as S = π (0.10)^2^
Insertion depth	3 cm	Field measured
Chamber height above soil	12 cm	Field measured
Headspace volume (V)	0.00377 m^3^	Calculated as V = πr^2^h, r = 0.10 m, h = 0.12 m
Material	PLA filament (generic supplier, Shenzhen, China)	3D printed
Wall thickness	2 mm	Structural design choice
Sealing method	Epoxy + grommets	Sealing of tubing and cable exits

**Table 2:** Material costs.

Component	Unit Cost (USD)	Quantity	Total Cost (USD)
Raspberry Pi 4	$35	1	$35
MH-Z19 CO_2_ Sensor	$25	1	$25
LM393 Moisture Sensor	$7	1	$7
Soil Temperature Sensor	$10	1	$10
3D Printed Chamber (PLA)	$20	1	$20
Wires & Connectors	$5	1	$5
ADC1115 (ADC)	$15	1	$15
USB to TTL	$10	1	$10
Pump Motor	$10	1	$10
Batteries (9V)	$5	2	$10
Buttons	$5	1	$5
Relay Module	$10	1	$10
Total			$162

**Table 3:** Side-by-side comparison settings for soil CO_2_ flux measurements.

Parameter	Low-Cost Chamber	LI-COR 8200-01S
Site	Corn field, Prairie View A&M University, TX	Corn field, Prairie View A&M University, TX
Soil condition	Agricultural soil	Agricultural soil
Collar inner diameter	20 cm	20 cm
Distance between chambers	2–5 cm	2–5 cm
Number of measurement rounds	Multiple repeated rounds	Multiple repeated rounds
Time per round	120 s chamber closure	Standard LI-COR automated cycle
Flux calculation method	Linear slope of CO_2_ vs. time	LI-COR internal algorithm

## Data Availability

The datasets generated and/or analyzed during the current study are available from the corresponding author upon reasonable request.
